# Differential gene expression following TLR stimulation in *rag1^-/-^* mutant zebrafish tissues and morphological descriptions of lymphocyte-like cell populations

**DOI:** 10.1371/journal.pone.0184077

**Published:** 2017-09-14

**Authors:** Preeti J. Muire, Larry A. Hanson, Robert Wills, Lora Petrie-Hanson

**Affiliations:** 1 Department of Basic Sciences, College of Veterinary Medicine, Mississippi State University, Mississippi State, Mississippi, United States of America; 2 Department of Pathobiology and Population Medicine, College of Veterinary Medicine, Mississippi State University, Mississippi State, Mississippi, United States of America; University of Miami, UNITED STATES

## Abstract

In the absence of lymphocytes, *rag1*^*-/-*^ mutant zebrafish develop protective immunity to bacteria. In mammals, induction of protection by innate immunity can be mediated by macrophages or natural killer (NK) cells. To elucidate potential responsive cell populations, we morphologically characterized lymphocyte-like cells (LLCs) from liver, spleen and kidney hematopoietic tissues. In fish, these cells include NK cells and Non-specific cytotoxic cells (NCCs). We also evaluated the transcriptional expression response of select genes that are important indicators of NK and macrophage activation after exposure to specific TLR ligands. The LLC cell populations could be discriminated by size and further discriminated by the presence of cytoplasmic granules. Expression levels of *mx*, *tnfα*, *ifnγ*, *t-bet* and *nitr9* demonstrated dynamic changes in response to intra-coelomically administered β glucan (a TLR2/6 ligand), Poly I:C (a TLR3 ligand) and resiquimod (R848) (a TLR7/8 ligand). Following TLR 2/6 stimulation, there was a greater than 100 fold increase in *ifnγ* in liver, kidney and spleen and moderate increases in *tnfα* in liver and kidney. TLR3 stimulation caused broad up regulation of *mx*, down-regulation of *tnfα* in kidney and spleen tissues and up regulation of *nitr9* in the kidney. Following TLR 7/8 stimulation, there was a greater than 100 fold increase in *ifnγ* in liver and kidney and *t-bet* in liver. Our gene expression findings suggest that LLCs and macrophages are stimulated following β glucan exposure. Poly I:C causes type I interferon response and mild induction of LLC in the kidney and R-848 exposure causes the strongest LLC stimulation. Overall, the strongest NK like gene expression occurred in the liver. These differential effects of TLR ligands in *rag1*^*-/-*^ mutant zebrafish shows strong NK cell-like gene expression responses, especially in the liver, and provides tools to evaluate the basis for protective immunity mediated by the innate immune cells of fish.

## Introduction

Innate immunity provides essential protection against pathogens during early life stages of fish because adaptive immunity is absent [1–3]. Zebrafish develop adaptive immunity 3 weeks post fertilization [4]. *Rag1*^*-/-*^ mutant zebrafish lack T cell receptor (TCR) and immunoglobulin (Ig) but have non-specific cytotoxic cells (NCCs), NK cells, monocytes/macrophages and neutrophils [5]. Using these fish as a model for studying immune responses in young fish, we found that fish lacking effector lymphocytes can develop protective immunity to bacteria after vaccination [6]. However, the specific cell population(s) mediating this protection has not been identified. Unfortunately, tools for use in fish immunity research are very few [7], so separating immune cell populations in fish species is very challenging. Research in mammalian systems suggest that Natural Killer cells and macrophages can mediate a level of protection [8]. Recent studies using viral pathogens in *rag1*^*-/-*^ mutant zebrafish showed enhanced NK and macrophage specific gene expression [9] as defined in earlier transcriptome analysis [10]. To determine the cell populations involved in our model, we used a transcriptome study to compare gene expression after the primary response to gene expression after the secondary protective response. Genes for cell receptor activation and signal transduction, cell proliferation and cytotoxic functions were up-regulated. These findings suggested receptor activation and expansion of a cell population. Increased *ifn*γ expression was associated with both primary and secondary immune responses [11]. These findings suggested functional responses of cell division and enhanced phagocytosis, but did not indicate a specific cell population was associated with the protective secondary response.

To better characterize the cell populations that may mediate protective immunity in our zebrafish model, we evaluated gene expression responses associated with specific pathogen recognition processes of innate immune cells. These cells have a variety of pattern recognition receptors (PRRs) that allow them to respond quickly to pathogens based on certain conserved pathogen associated molecular patterns (PAMPs). Toll like receptors (TLRs) are among the best-characterized PRRs and are present on macrophages, B cells, dendritic cells and NK cells. They have important roles in mediating innate and adaptive immune responses. Twenty-two TLRs have been documented in zebrafish [12].

We used β glucan, Poly I:C and R848 as model PAMPs. β glucan stimulates multiple cell types, while poly I:C and R849 are documented to stimulate NK cells more specifically (12,17–20,22,27). β glucan is a yeast cell wall derivative that specifically binds to TLR2/6 [13–18]. Recognition of β glucan by the TLR2/6 heterodimer requires Dectin-1 as a co-receptor [19]. Previous studies have shown that β glucan triggers the activation of macrophages, neutrophils, monocytes, NK cells and dendritic cells and can enhance the function of human NK cell cytotoxicity [16].

Poly I:C is a synthetic dsRNA molecule that binds to endosomal TLR3 [20]. It is one of the most commonly studied TLR ligands, and stimulates type I interferon and *mx* production [20] and is known to activate mammalian NK cells in the absence of antigen presenting cells [21–24]. Poly I:C induced *mx* expression and demonstrated anti-viral activity in flatfish [25]. Further, in mice, Poly I:C treatment induced the activation and accumulation of hepatic NK cells [22, 26].

R848 is a synthetic compound that mimics viral ssRNA. It is a ligand of endosomal TLR7/8 and belongs to the family of imidazoquinolines, that are known to induce interferon expression [27–30], especially *ifnγ* expression from mammalian NK cells [31].

To evaluate PAMP recognition by innate immune cells, we evaluated key immune response genes *mx*, *tnfα*, *ifnγ*, *t-bet* and *nitr9*. *Mx* is an indirect indicator of type I interferon expression in teleosts [20, 32–36] and can inhibit viral replication at various stages of the virus life cycle [25]. *Tnfα* is a pro-inflammatory cytokine critical to the host during bacterial and viral infections [37, 38], and is an indicator of NK cell and macrophage and dendritic cells stimulation. *Ifnγ* is a critical proinflammatory cytokine required for protection against bacterial and viral attacks [32, 39–42]. Teleosts NK and T cells produce *ifnγ* [32]. *Tbox-21* (*t-bet*) is a transcription factor required for development of mammalian NK and Th1 cells [43]. *Nitr9* is a putative activating receptor with immunoreceptor tyrosine-based activation motifs (ITAMs), similar to mammalian activating NK receptors (KIRs and Ly49s) [44]. In this study, expression of the chosen immune response genes provides information on relative stimulation of general induction: *mx*, macrophage induction: *tnfα*, and NK cell induction: *ifnγ*, *t-bet* and *nitr9*. We used these genes and the T and B cell deficient *rag*1^*-/-*^ mutant zebrafish model to discern the potential contribution of innate immune cell populations following PRR stimulation. In this study we evaluated temporal tissue specific transcript expression of immune relevant genes of *rag*1^*-/-*^ mutant zebrafish in response to TLR ligands so that the process of PAMP recognition by LLC populations could be evaluated. We also morphologically characterized LLCs isolated from liver, kidney and spleen tissues of these fish.

## Materials and methods

### 2.1 *Rag1*^*-/-*^ mutant zebrafish care

All zebrafish used in this study were bred from a homozygous colony of *rag1*^*-/-*^ mutant zebrafish previously established in the specific pathogen free hatchery in the College of Veterinary Medicine, Mississippi State University (MSU) [5]. Propagation and experimental protocols were approved by the MSU Institutional Animal Care and Use Committee (IACUC).

### 2.2 Cell isolations, cytospins and flow cytometry

Liver, kidney and spleen tissues were removed from 3 *rag1*^*-/-*^ mutant zebrafish and individually weighed. Each tissue type from 3 fish were pooled and comprised one replicate. The weight of each tissue was recorded. Tissues were collected in cold FACS buffer (2% BSA in Hank’s buffer) and disrupted on ice with a teflon homogenizer. The homogenate was passed through a sterile 40μm nylon cell strainer to make single cell suspensions. The liver sample was passed through the cell strainer twice to remove cell clumps. Filtered cells were placed on a histopaque 1119 gradient (Sigma–Aldrich) and the buffy layer collected. This suspension was centrifuged at 400xg for 20 minutes, and the supernatant decanted off. Pellets from the kidney and liver were resuspended in 2.5 mls and the spleen pellet was resuspended in 1 ml of cold Hank’s buffer without Ca^2+^ and Mg^2+.^ Cells were counted with a BioRad TC20^™^ Automated Cell Counter and viability was assessed by Trypan blue (Invitrogen) exclusion.

The cytospin cartridges were filled with 400μl of cell suspension containing 10^4^ to 10^6^ cells/ml and centrifuged in a Cyto-tech^®^ centrifuge at 500 x g for 1 minute. The cytospin slides were air dried for 20 minutes and stained with Wrights-Giemsa stain (Fisher Scientific Company) following the manufacturer’s instructions. Slides were viewed on an Olympus BX43 at 1000x magnification. Differential leukocyte counts were performed in each tissue. Cell classifications were based on morphology. Lymphocyte-like cells (LLCs) were classified as small, large, and agranular or granular.

Flow cytometry procedures were carried out as previously performed in our lab [5]. Briefly, each cell preparation was transferred into 3 mL tube containing 2 mL phosphate buffered saline with 1% fetal bovine serum, Sigma-Aldrich, St. Louis, MI. Zombie green cell viability dye (Biolegend #423111) was used to determine cell survival. Cells were kept on ice until analyzed by forward scatter and side scatter on a FACS Calibur (Becton Dickinson). 20,000 cells were collected per tissue. Forward scatter (FSC) represents cell diameter. The running parameters were amp gain 3.0 and the threshold 80. Side scatter (SSC) represents cell granularity or complexity. The amp gain was 1.0 and the threshold was 80.

### 2.3 Quantifying gene expression

*Rag1*^*-/-*^ zebrafish were injected intra-coelomically (IC) with β glucan (50μg/0.5g of fish), Poly I:C (50μg/0.5g of fish) and R848 (0.08μl/0.5g of fish) or endotoxin free PBS (10μl/fish). Fish were euthanized in buffered 0.02% MS222 and liver, kidney and spleen tissues from fish were excised at 0h (non-injected fish (n = 5) for basal expression), 1, 6, 12 and 24 hours post injection (hpi) (n = 3) for each TLR ligand. Whole tissues were immediately transferred to 400μl Trizol reagent (Zymo Research, USA) and homogenized following standard procedures in our lab [45]. Total RNA was extracted from each liver, kidney and spleen sample using RNA extraction kits (Zymo Research, USA) according to the manufacturer’s protocol. The quantity of extracted total RNA was determined by NanoDrop ND-1000 and ND-8000 8-Sample Spectrophotometer and stored at -80°C until used. cDNA was prepared from 100ng of RNA using Super script III VILO^™^ cDNA Synthesis Kit (Invitrogen).

*Mx*, *tnfα*, *ifnγ*, *t-bet* and *nitr9* were measured using real time quantitative PCR. The *mx* and *t-bet* primers and probes (Table 1) were designed by Beacon Design software (BioRad) and Primer3 plus (GraphPad) software, respectively. The source of the other primers and probes are included in Table 1. All primers and probes were purchased from Eurofins MWG, Operon, Huntsville, Alabama, USA. Amplification of the ubiquitously expressed acidic ribosomal phosphoprotein (*arp*) gene was used for the internal control [46]. The amplification was performed in a 25μl volume containing 10 μl target cDNA and 15 μl master mix containing: 8.8 μl Nuclease free water (GIBCO, Ultra Pure^™^), 1.5 μl MgCl_2_ (5mM), 2.5 μl 10x buffer, 0.5 μl dNTPs, 0.2μl Taq Polymerase HS enzyme (Hot Start PCR Kit, TAKARA, Japan), 0.5 μl forward primer (20μM), 0.5 μl reverse primer (20μM) and 0.5 μl probe (10μM). Thermal cycler parameters for the PCR program were set as follows: 50°C for 2 minutes, 95°C for 10 minutes, 45 cycles of 95°C for 15 seconds and 61°C for 1 minute. All samples (biological reps) were run in triplicates i.e., 3 technical reps/sample.

**Table 1:** Oligonucleotide primers and probes used for qRT-PCR to quantify gene expression levels of *mx*, *tnfα*, *ifnγ*, *t-bet* and *nitr9*.

Gene	Oligonucleotide sequences (5’–3’)	GenBank Accession No.
***arp***	**Fwd:**CTGCAAAGATGCCCAGGGA	NM_131580
	**Rev:**TTGGAGCCGACATTGTCTGC	
	**Probe:**[6~FAM]TTCTGAAAATCATCCAACTGCTGGATGACTACC [BHQ1a~ Q] [47]	
***mx***	**Fwd:**GCATCATTAGTTCAGACAGTCG	NM_182942.4
	**Rev:**AAATTATCGATAGTGTCGATACAAG	
	**Probe:**[6~FAM]TGCTGACTGAACGTGTAACTCAACT [BHQ1a~ Q] *	
***tnfα***	**Fwd:**TCGCATTTCACAAGGCAATTT	NM_212859
	**Rev:**GGCCTGGTCCTGGTCATCTC	
	**Probe:**[6~FAM]AGGCTGCCATCCATTTAACAGG[BHQ1a~Q] [47]	
***ifnγ***	**Fwd:**CTTTCCAGGCAAGAGTGCAGA	NM_212864
	**Rev:**TCAGCTCAAACAAAGCCTTTCG	
	**Probe:**[6~FAM]AACGCTATGGGCGATCAAGGAAAACGAC[BHQ1a~ Q] [47]	
***t-bet***	**Fwd:**GATCAAGCTCTCTCTGTGATAG	NM_001170599.1
	**Rev:**GCTAAAGTCACACAGGTCT	
	**Probe:**[6~FAM]TTCTGAAGGTCACGGTCACA[BHQ1a~Q] *	
***nitr9***	**Fwd:**GTCAAAGGGACAAGGCTGATAGTT	AY570237.1
	**Rev:**GTTCAAAACAGTGCATGTAAGACTCA	
	**Probe:** [6~FAM]CAAGGTTTGGAAAAGCAC[BHQ1a~Q] [48]	

Housekeeping gene *arp* (house-keeping gene) was used as a reference gene.

* The *mx* and *t-bet* primers and probes were designed by Beacon Design software (BioRad) and Primer3 plus (GraphPad) software, respectively.

### 2.4 Quantifying protein expression

*Rag1*^*-/-*^ mutant zebrafish were injected with R848 (0.08μl /fish) and the liver, kidney and spleen were sampled at 6 hpi. Tissues were homogenized in tissue protein extraction buffer (T-PER) (ThermoScientific) and supernatant was collected. Protein concentration was estimated in the supernatant by Bradford’s assay (Sigma-Aldrich). 30mg of protein from liver, kidney and spleen cell lysate was resolved on 12% SDS-polyacrylamide gels and transferred to polyvinylidene difluoride (PVDF) membranes (BioRad) for western blot analysis. Poly vinyl d f (PVDF) membranes were incubated in blocking buffer (3% milk in Tris-buffered saline with 0.1% Tween 20 (TBS-T)) overnight. Membranes were washed in TBS-T and TBS twice for 5 minutes and once for 5 minutes respectively and were incubated with primary antibody: IgG mouse anti zebrafish Nitr9^90.10.5^ monoclonal antibody (1:500), a gift from J. Yoder (Shah et al 2012). Membrane was washed in TBST and TBS twice for 5 minutes and once for 5 minutes respectively and incubated with secondary antibody: goat anti-mouse IgG/HRP conjugated (1:4000) (ThermoScientific) for 1h at 4°C. The PVDF blot was stripped for 7 minutes at room temperature by Restore^™^ PLUS Western Blot Stripping buffer (ThermoScientific) followed by one wash in TBS for 5 minutes. Membrane was incubated in blocking buffer overnight at 4°C followed by incubation with anti-GAPDH rabbit polyclonal antibody (1:500) (AnaSpec, Fremont, CA) overnight at 4°C. Membrane was washed in TBST and TBS twice for 5 minutes and once for 5 minutes respectively and incubated with secondary antibody goat anti-rabbit IgG-HRP conjugated (1:2000) (ThermoScientific) for 1h at 4°C. To visualize the bands, PVDF membrane was washed in TBST and stained with Pierce ECL western blotting substrate (ThermoScientific) and developed by using clear blue X-Ray Film (ThermoScientific). Band densities were determined using Studio Lite Software (Li-Cor).

### 2.5 Data analysis and statistical evaluation

The different cell sizes were averaged and a Student’s t-test statistical analyses was preformed to compare the cell size within each tissue. ANOVA was performed to compare cell sizes between tissues. An alpha level of 0.05 was used to determine the significance of all analyses. Flow cytometry analyzed cells by forward scatter (FSC) and side scatter (SSC) properties.

Relative gene expression was determined using the Pfaffl method [49]. Data obtained from qRT-PCR were expressed as fold change and were converted to log2 values. Data were analyzed by two-way analysis of variance using PROC MIXED (SAS for Windows 9.4, SAS Institute, Inc., Cary, NC) and are shown in S1 Table. Separate models were used for each gene and tissue combination. The explanatory variables for all models were treatment, time post injection, and the treatment x time interaction. The effect of treatment was reported only if the treatment and treatment x time interaction was significant. If the interaction was significant, treatment to control comparisons were made at each time point with p values corrected for multiple comparisons using the SIMULATE option in a LSMESTIMATE statement. Only treatments that resulted in gene expressions that were significantly different are represented graphically. Similarly, the 0 hour (non-injected fish) data were transformed to log2 values and analyzed by analysis of variance using PROC MIXED (SAS for Windows 9.4, SAS Institute, Inc., Cary, NC). Separate models were used for *mx*, *tnfα*, *ifnγ*, *t-bet* and *nitr9* to compare their basal expression within the liver, kidney and spleen. Significant differential gene expressions were summarized and placed into one of four categories: <10 fold change, 10–100 fold change, >100 fold change or down -regulated.

## Results

### 3.1 Morphological descriptions of lymphocyte-like cells (LLC) in the liver, kidney and spleen of T and B cell deficient *rag1*^*-/-*^ mutant zebrafish

**Fig 1:**
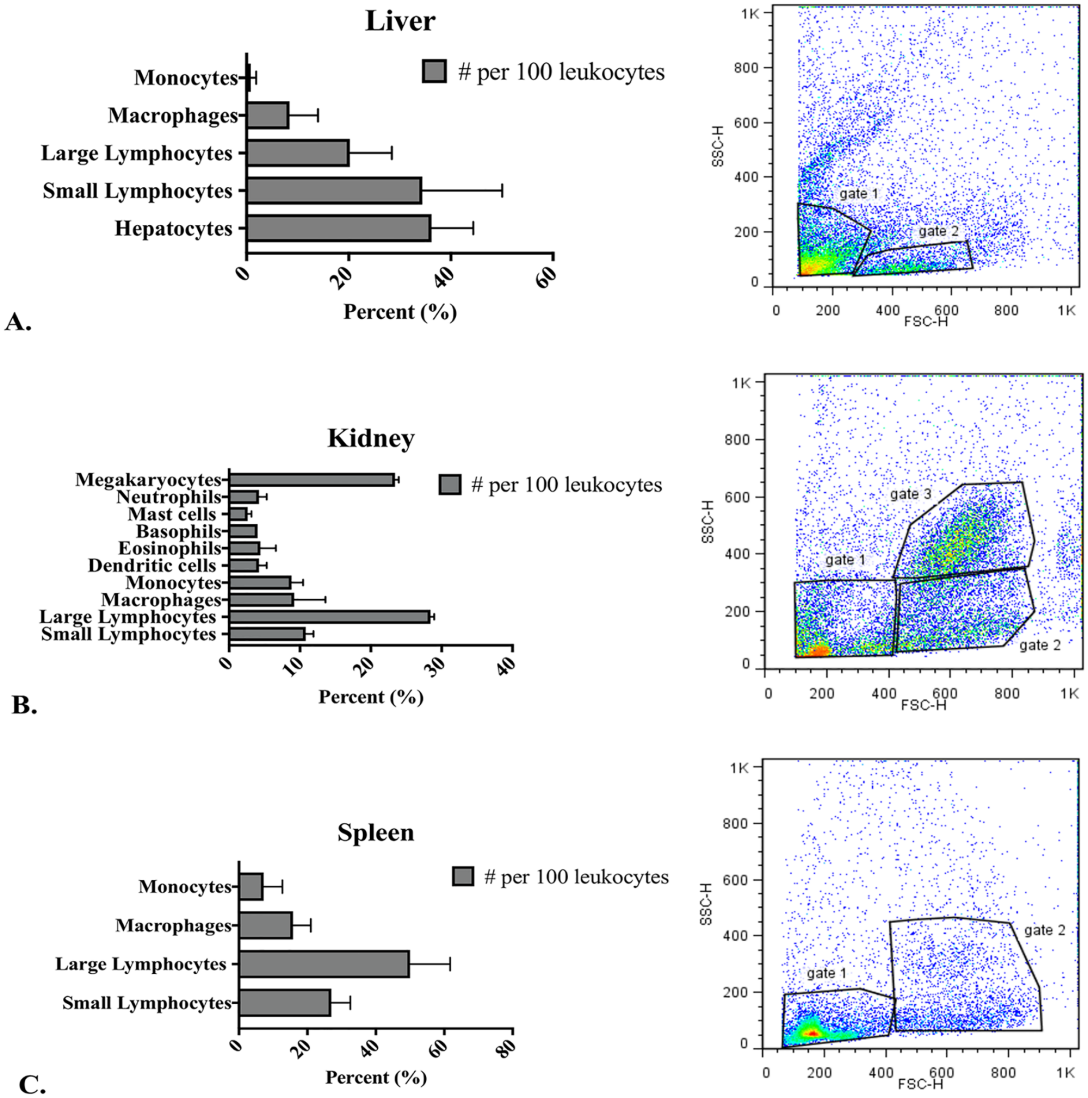
Leukocytes from liver (A), kidney (B) and spleen (C) tissues, based on morphological appearance and FACS forward scatter (FSC) and side scatter (SSC) characteristics.

The average weights of liver, kidney and spleen tissues were 43.33 mg, 15 mg and 2.66 mg respectively. The average number of cells isolated from the 1119 gradients were 1x 10^6^ cells from liver tissue, 6.75 x10^5^ cells from kidney tissue, and 1.4x10^5^ cells from spleen tissue. Leukocyte differentials revealed different predominate cell populations in liver, kidney and spleen tissues (Fig 1). Liver preparations were 36% hepatocytes, 34% small agranular LLCs, 20% large agranular LLCs and 12% macrophages/monocytes. Kidney preparations were 10% small agranular and granular LLCs, 28% agranular and granular large LLCs, 23% megakaryocytes, 15% granulocytes, 4% dendritic cells, 9% monocytes and 9% macrophages. Spleen preparations were 27% small agranular and granular LLCs, 50% large granular LLCs, 16% macrophages and 7% monocytes.

Examination of kidney, liver and spleen cells of the *rag1*^*-/-*^ mutant zebrafish by FACS analysis demonstrated different cell populations (Fig 1). On the basis of forward scatter (FSC) and side scatter (SSC) properties and location in a FSC vs. SCC plot, liver small agranular LCCs were included in gate 1 and large agranular LCCs were included in gate 2 (Fig 1A), the kidney small agranular and granular LCCs were included in gate 1 and large agranular and granular LCCs were included in gate 2 (Fig 1B), and macrophages, large granulocytes and megakaryocytes were included in gate 3. Spleen small agranular and granular LCCs were included in gate 1 and large granular LCCs were included in gate 2 (Fig 1C).

**Fig 2:**
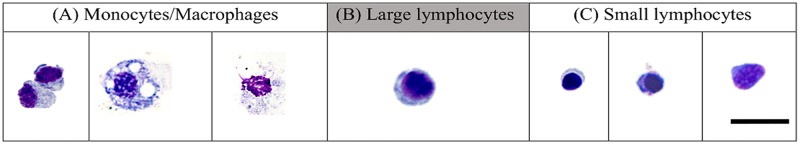
Leukocytes in *rag1*^*-/-*^ mutant zebrafish liver included: (A) monocytes/macrophages, (B) large LLCs, (C) small LLCs. Cells were stained with Wright Giemsa and examined under oil immersion by light microscopy and viewed at 1000x magnification. The size bar represents 10μm.

**Fig 3:**
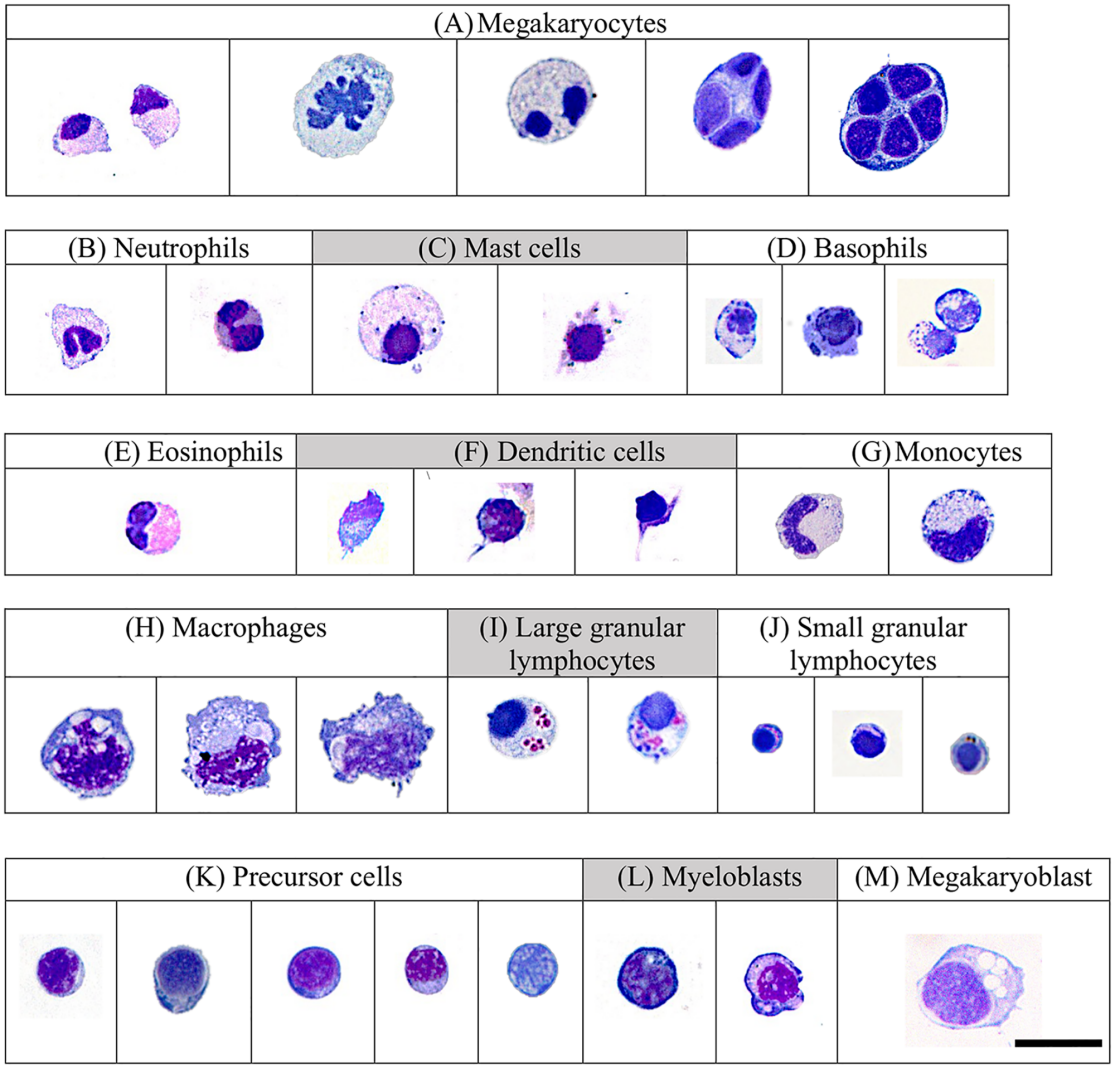
Leukocytes in *rag1*^*-/-*^ mutant zebrafish kidney included: (A) megakaryocytes, (B) neutrophils, (C) mast cells, (D) basophils, (E) eosinophils, (F) dendritic cells, (G) monocytes, (H) macrophages, (I) large granular LLCs, (J) small granular LLCs, (K) precursor cell, (L) myeloblasts and (M) megakaryoblast. Cells were stained with Wright Giemsa and examined under oil immersion by light microscopy and viewed at 1000x magnification. The size bar represents 10μm.

**Fig 4:**
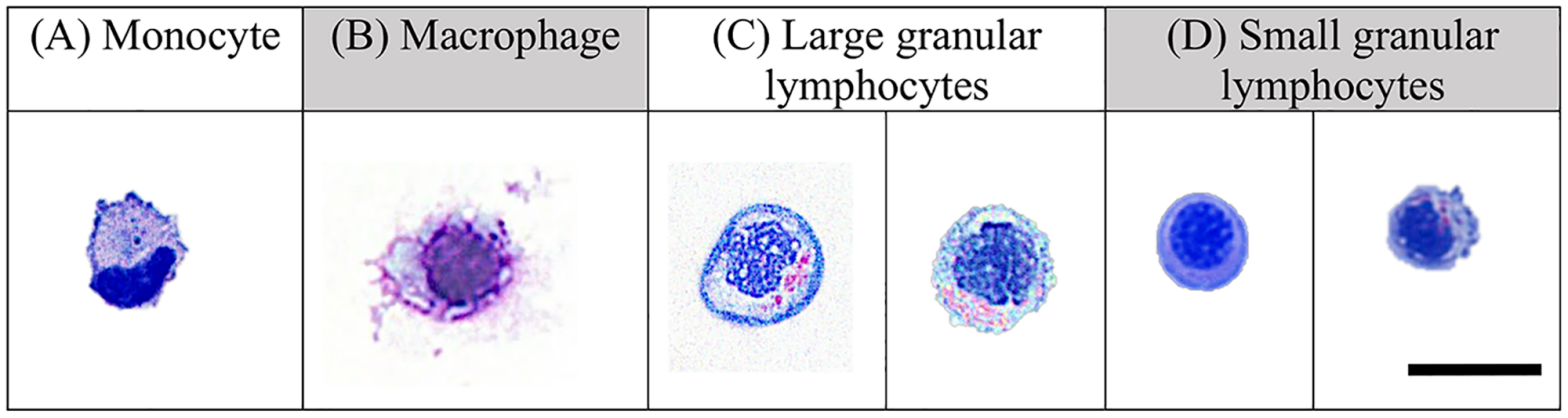
Leukocytes in *rag1*^*-/-*^ mutant zebrafish spleen included: (A) monocytes, (B) macrophages, (C) large granular and small granular and agranular LCCs. Cells were stained with Wright Giemsa and examined under oil immersion by light microscopy and viewed at 1000x magnification. The size bar represents 10μm.

Non-erythroid cytospin preparations from liver, kidney and spleen tissues revealed LLC populations with NK cell morphological characteristics (Figs 2–4). Two sizes of LCCs were seen (Table 2). Differences in granularity were observed in small and large cells. The ratio of large agranular, large granular, small agranular and small granular was different in liver, kidney and spleen tissues. In the liver (Fig 2), small agranular LCCs were 4.8± 0.8 μm and large agranular LCCs were 7.4 ± 1.1 μm. In the kidney (Fig 3), small LCCs were 6.2 ± 1.3 μm and large LCCs were 8.8 ± 1.1 μm. Both granular and agranular large LLCs and granular and agranular small LLCs were seen in the kidney. In the spleen (Fig 4), small 6.45 ± 0.5 μm and large 8.7 ± 1.2 μm LCCs were seen. Granular and agranular small LLCs and large granular LCCs were seen in the spleen. Within each tissue, the large cell size was significantly different from the small cell size. The size of the large cells was not significantly different between liver, kidney and spleen tissues. The size of the small cells was not significantly different between liver, kidney and spleen tissues.

**Table 2:** Size analyses of lymphocyte-like cells from liver, kidney and spleen tissues in *rag1*^*-/-*^ mutant zebrafish.

Tissues	Cells	Size ± SD	t-test p value	ANOVA
Liver	Large	7.4 ± 1.1 μm	0.0039*	A
	Small	4.8 ± 0.8 μm		B
Kidney	Large	8.8 ± 1.1 μm	0.0096*	A
	Small	6.2 ± 1.3 μm		B
Spleen	Large	9.76 ± 1.1 μm	0.0005*	A
	Small	6.45 ± 0.5 μm		B

Cells with the same letter are not significantly different from each other.

*significant when alpha value is <p value. (p = 0.005).

### 3.2 Differential gene expression

#### 3.2.1. Basal gene expression

No significant differences were observed in the gene expressions of *mx*, *tnfα*, *ifnγ*, *t-bet* and *nitr9* between different tissues prior to injecting *rag1*^*-/-*^ mutant zebrafish with TLR ligands (S1 Fig).

#### 3.2.2. Effect of β glucan on *mx*, *tnfα*, *ifnγ*, *t-bet* and *nitr9* expression in liver, kidney and spleen

β glucan did not affect *mx* expression in the liver, kidney or spleen. Statistical values for tissue gene expression studies were summarized (S1 Table).

**Fig 5:**
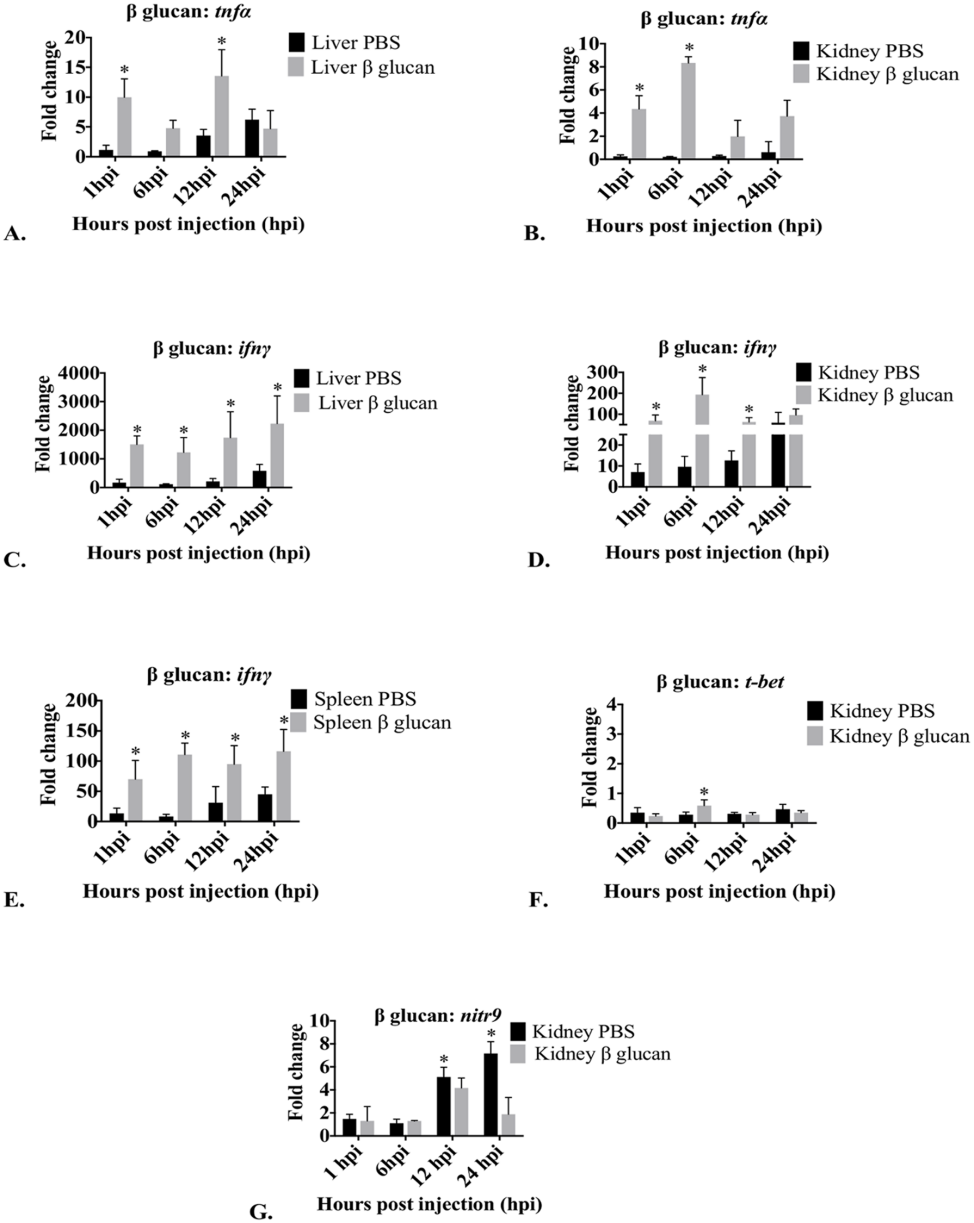
Graphs depicting changes in mRNA expression over time of *tnfα*, *ifnγ*, *t-bet* and *nitr9* in liver, kidney and spleen after treatment with β glucan. Only tissues and genes that demonstrated significant changes in expression compared to PBS injected controls are presented. Fold changes in *tnfα* in liver (A) and kidney (B), *ifnγ* in liver (C), kidney (D) and spleen (E), *t-bet* in kidney (F) and *nitr9* in kidney (G) are presented as mean fold change relative to the time zero group ± standard deviation as measured by quantitative RT-PCR. *Arp* was used as a housekeeping gene. hpi = hours post injection; control = PBS (endotoxin-free); Treated = β glucan. *Significant (p<0.05) difference in expression of treated compared to control. No significant changes in expression were observed in *tnfα* in spleen, in *t-bet* in liver and spleen and in *nitr9* in liver and spleen (S1 Table).

Liver *tnfα* expression was affected by β glucan and expression was significantly different between time points (Fig 5A). β glucan significantly increased the expression of *tnfα* at 1 hpi and 12 hpi but not at 6 hpi and 24 hpi. In the kidney, *tnfα* expression was significantly different between time points (Fig 5B). β glucan caused significantly greater *tnfα* expression at 1 hpi and 6 hpi but not at 12 hpi or 24 hpi. In the spleen, *tnfα* expression was not significantly affected (S1 Table).

In the liver, *ifnγ* expression was significantly affected by β glucan exposure at 1 hpi, 6 hpi, 12 hpi and 24 hpi (Fig 5C). In the kidney, expression was significantly different between time points (Fig 5D). Kidney *ifnγ* expression was significantly greater at 1 hpi, 6 hpi and 12 hpi. β glucan did not significantly increase *ifnγ* at 24 hpi. In the spleen, *ifnγ* expression was significantly affected by β glucan at 1 hpi, 6 hpi, 12 hpi and 24 hpi (Fig 5E).

*T-bet* expression in the liver was not significantly affected by β glucan. In the kidney, expression was significantly different between time points (Fig 5F), with *t-bet* expression significantly greater only at 6 hpi. β glucan did not significantly affect *t-bet* expression at 1 hpi, 12 hpi or 24 hpi. In the spleen, expression of *t-bet* was not significantly affected by β glucan.

β glucan had no effect on *nitr9* expression in the liver. In the kidney, expression of *nitr9* was significantly different between time points (Fig 5G), with *nitr9* expression significantly down-regulated at 12 hpi and 24 hpi. β glucan had no effect on *nitr9* expression in the spleen.

#### 3.2.3. Effect of Poly I:C on *mx*, *tnfα*, *ifnγ*, *t-bet* and *nitr9* in liver, kidney and spleen

**Fig 6:**
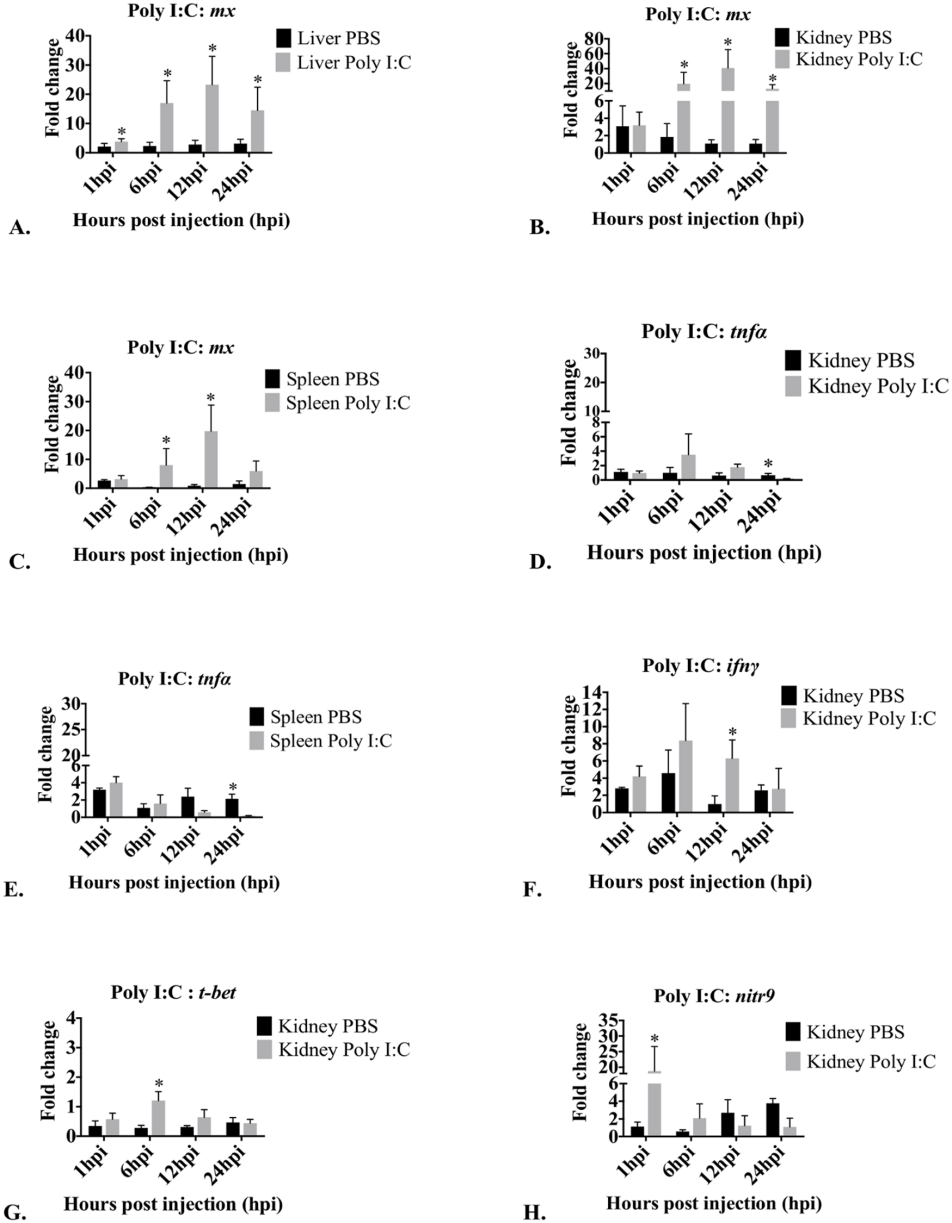
Graphs depicting changes in mRNA expression over time of *mx*, *tnfα*, *ifnγ*, *t-bet* and *nitr9* in liver, kidney and spleen after treatment with Poly I:C. Only tissues and genes that demonstrated significant changes in expression compared to PBS injected controls are presented. Fold changes in *mx* in liver (A), kidney (B) and spleen (C), *tnfα* in kidney (D) and spleen (E), *ifnγ* in kidney (F), *t-bet* in kidney (G) and *nitr9* in kidney (H) are presented as mean fold change relative to the time zero group ± standard deviation as measured by quantitative RT-PCR. *Arp* was used as a housekeeping gene. hpi = hours post injection; control = PBS (endotoxin-free); Treated = Poly I:C. *Significant (p<0.05) difference in expression of treated compared to control. No significant changes in expression were observed in *tnfα* in liver, in *ifnγ*, *t-bet* and in *nitr9* in liver and spleen (S1 Table).

Poly I:C significantly affected *mx* expression in liver at 1 hpi, 6 hpi, 12 hpi and 24 hpi (Fig 6A). In the kidney, expression was significantly different between time points (Fig 6B). There was increased *mx* expression at 6 hpi, 12 hpi and 24 hpi. Kidney *mx* expression was not effected by Poly I:C at 1 hpi. In the spleen, expression was significantly different between time points (Fig 6C). The expression of *mx* was significantly greater at 6 hpi and 12 hpi. Poly I:C did not significantly affect *mx* expression at 1 hpi and 24 hpi.

Liver *tnfα* expression was not significantly affected by Poly I:C. In the kidney, expression was significantly different between time points (Fig 6D). The expression of *tnfα* was significantly down-regulated at 24 hpi. In the spleen, the expression between different time points was significantly different, and *tnfα* expression was significantly down-regulated at 24 hpi (Fig 6E).

Poly I:C did not affect *ifnγ* expression in the liver. In the kidney, expression was significantly different between time points (Fig 6F), and *ifnγ* expression was significantly greater at 12 hpi. Poly I:C did not significantly affect kidney *ifnγ* expression at 1 hpi, 6 hpi and 24 hpi. Poly I:C did not significantly affect splenic expression of *ifnγ* (S1 Table).

Poly I:C had no effect on *t-bet* expression in the liver. In the kidney, expression was significantly different between time points (Fig 6G), and Poly I:C significantly increased *t-bet* expression at 6 hpi. However, Poly I:C did not affect *t-bet* expression at 1 hpi, 12 hpi and 24 hpi. Poly I:C had no effect on *t-bet* expression in the spleen.

Poly I:C did not significantly affect *nitr9* expression in liver. In the kidney, *nitr9* expression was significantly different between time points (Fig 6H), and *nitr9* expression was significantly greater at 1 hpi. Poly I:C did not affect *nitr9* expression at 6 hpi, 12 hpi and 24 hpi. Poly I:C did not affect splenic *nitr9* expression.

#### 3.2.4. Effect of R-848 on *mx*, *tnfα*, *ifnγ*, *t-bet* and *nitr9* in liver, kidney and spleen

**Fig 7:**
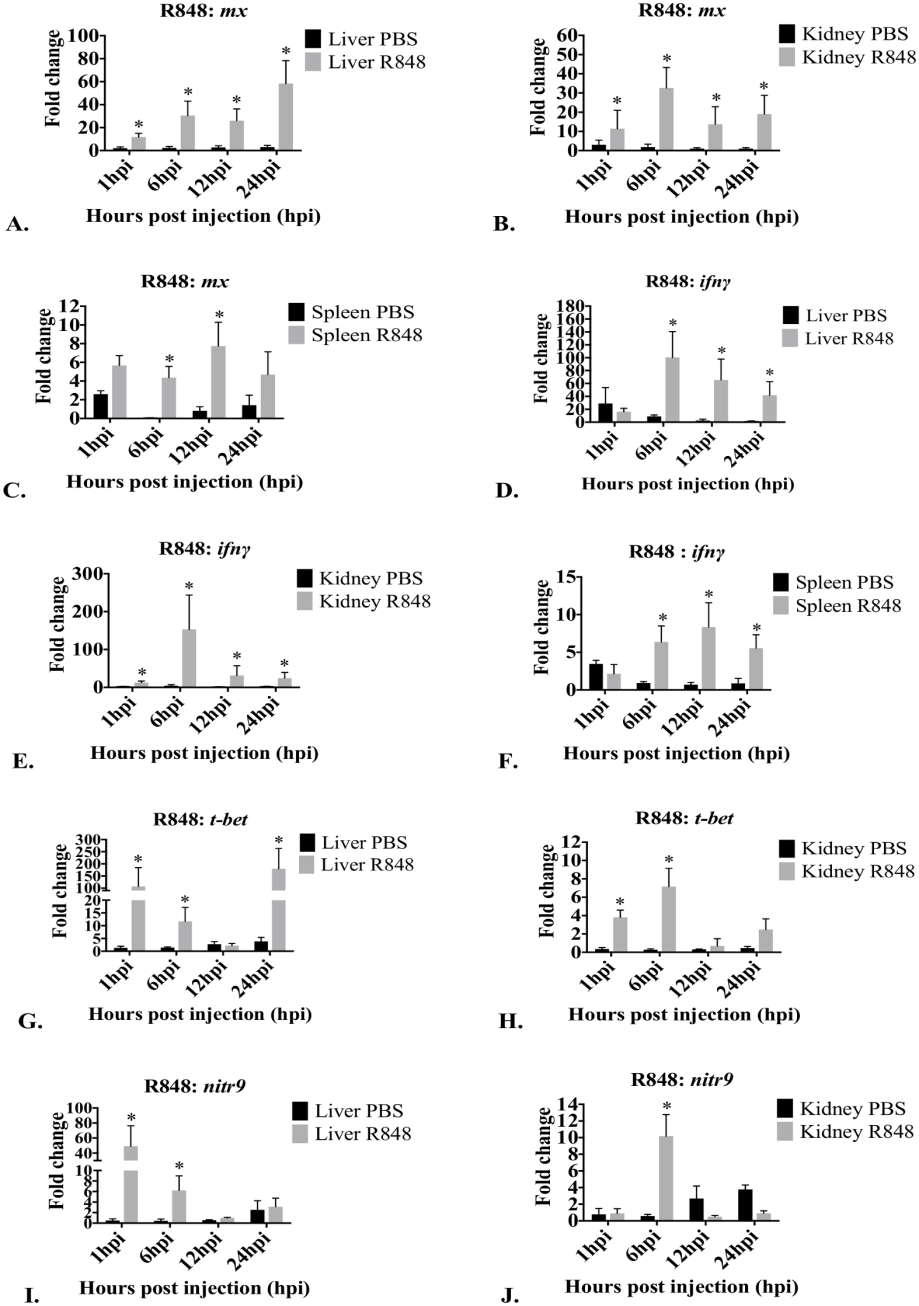
Graphs depicting changes in mRNA expression over time of *mx*, *tnfα*, *ifnγ*, *t-bet*, and *nitr9* in liver, kidney and spleen after treatment with R848. Only tissues and genes that demonstrated significant changes in expression compared to PBS injected controls are presented. Fold changes in *mx* in liver (A), kidney (B), and spleen (C), *ifnγ* in liver (D), kidney (E) and spleen, (F), *t-bet* in liver (G), and kidney (H) and *nitr9* in liver (I) and kidney (J) are presented as mean fold change relative to the time zero group ± standard deviations measured by quantitative RT-PCR. *Arp* was used as a housekeeping gene. hpi = hours post injection; control = PBS (endotoxin-free); Treated = R848. *Significant (p<0.05) difference in expression of treated compared to control. No significant changes in expression were observed in *t-bet* and *nitr9* in spleen (S1 Table).

Liver *mx* expression was significantly affected by R848 at 1 hpi, 6 hpi, 12 hpi and 24 hpi (Fig 7A). *Mx* expression was significantly affected by R848 in kidney at 1 hpi, 6 hpi, 12 hpi and 24 hpi (Fig 7B). Splenic *mx* expression was affected by R848 and expression was significantly different between time points (Fig 7C). R848 significantly increased the splenic *mx* expression at 6 hpi and 12 hpi. R848 did not affect splenic *mx* expression at 1 hpi and 24 hpi. R848 did not affect *tnfα* expression in the liver, kidney or spleen (S1 Table).

Liver *ifnγ* expression was affected by R848, and expression was significantly different between time points (Fig 7D). R848 significantly increased the expression of *ifnγ* at 6 hpi, 12 hpi and 24 hpi. However, R848 did not affect *ifnγ* expression at 1 hpi. In the kidney, *ifnγ* expression was affected by R848 at 1 hpi, 6 hpi, 12 hpi and 24 hpi (Fig 7E). Splenic *ifnγ* expression was affected by R848 and expression was significantly different between time points (Fig 7F). R848 significantly increased the expression of *ifnγ* at 6 hpi, 12 hpi and 24 hpi. However, R848 did not affect *ifnγ* expression at 1 hpi.

Liver *t-bet* expression was significantly different between time points (Fig 7G) and was increased at 1 hpi, 6 hpi and 24 hpi. R848 did not affect *t-bet* expression at 12 hpi. Kidney *t-bet* expression was significantly different between time points (Fig 7H) and was increased at 1 hpi and 6 hpi. R848 did not affect *t-bet* expression at 12 hpi and 24 hpi. In the spleen, expression of *t-bet* was not significantly effected by R848.

Liver *nitr9* expression was significantly different between time points (Fig 7I). R848 significantly increased *nitr9* expression at 1 hpi and 6 hpi. R848 did not affect *nitr9* expression at 12 hpi or 24 hpi. In the kidney, *nitr9* expression was significantly different between time points (Fig 7J), and was increased at 6 hpi. R848 did not affect *nitr9* expression at 1 hpi, 12 hpi and 24 hpi. In the spleen, *nitr9* expression was not significantly affected by R848.

### 3.3 Protein expression

**Fig 8:**
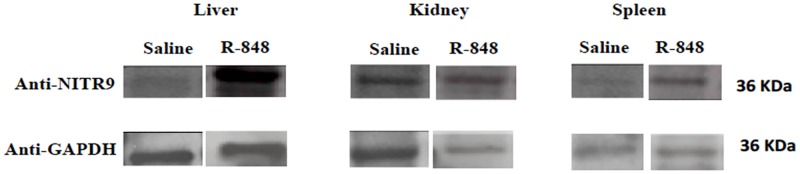
Images of western blots demonstrating NITR9 expression in the liver, kidney and spleen in *rag 1*^-/-^ zebrafish that were IC injected with R-848 or saline. The lower images demonstrate GAPDH expression on the same blot. The relative density (bottom) is the pixel density of the NITR9 band divided by the GAPDH.

Expression of NITR9 using anti-NITR9^90.10.5^ antibody in liver, kidney, and spleen tissues of *rag1*^*-/-*^ mutant zebrafish by western blot demonstrated higher expression in the liver and kidney following R848 treatment at 6 hours (Fig 8). These findings correlated with significantly up-regulated *nitr9* expression in the liver and kidney at 6 hpi of R848 (Table 3). Expression of NITR9 using anti-NITR9^90.10.5^ antibody in the spleen at the same time and treatment is increased, but not substantially. Both NITR9 and GAPDH are 36 KDa. Western blots band widths were by Image Studio Lite software (LI-COR).

**Table 3:** Summary of expression changes of significantly up-regulated and down-regulated genes at different hours post injection (hpi) following immune stimulation by β glucan, Poly I:C and R848 in liver, kidney and spleen of *rag1*^*-/-*^ mutant fish.

Treatment	Tissues	<10 fold change	10–100 fold change	>100 fold change	Down regulated genes
**β glucan**	**Liver**	*tnfα* (1 hpi)		*ifnγ* (1 hpi)	
		*tnfα* (12 hpi)		*ifnγ* (6 hpi)	
				*ifnγ* (12 hpi)	
				*ifnγ* (24 hpi)	
	**Kidney**	*tnfα* (1 hpi)	*ifnγ* (1 hpi)	*ifnγ* (6 hpi)	
		*tnfα* (6 hpi)	*ifnγ* (12 hpi)		
		*t-bet* (6 hpi)			
	**Spleen**		*ifnγ* (1 hpi)	*ifnγ* (6 hpi)	
			*ifnγ* (12 hpi)	*ifnγ* (24 hpi)	
**Poly I:C**	**Liver**	*mx* (1 hpi)			
		*mx* (6 hpi)			
		*mx* (12 hpi)			
		*mx* (24 hpi)			
	**Kidney**	*ifnγ* (12 hpi)	*mx* (6 hpi)		*tnfα* (24 hpi)
		*t-bet* (6 hpi)	*mx* (12 hpi)		
			*mx* (24 hpi)		
			*nitr9* (1 hpi)		
	**Spleen**	*mx* (6 hpi)	*mx* (12 hpi)		*tnfα* (24 hpi)
**R848**	**Liver**	*nitr9* (6 hpi)	*ifnγ* (12 hpi)	*ifnγ* (6 hpi)	
			*ifnγ* (24 hpi)	*t-bet* (1 hpi)	
			*nitr9* (1 hpi)	*t-bet* (24 hpi)	
			*t-bet* (6 hpi)		
			*mx* (1 hpi)		
			*mx* (6 hpi)		
			*mx* (12 hpi)		
			*mx* (24 hpi)		
	**Kidney**	*t-bet* (1 hpi)	*ifnγ* (1 hpi)	*ifnγ* (6 hpi)	
		*t-bet* (6 hpi)	*ifnγ* (12 hpi)		
			*ifnγ* (24 hpi)		
			*nitr9* (6 hpi)		
			*mx* (1 hpi)		
			*mx* (6 hpi)		
			*mx* (12 hpi)		
			*mx* (24 hpi)		
	**Spleen**	*mx* (6 hpi)			
		*mx* (12 hpi)			
		*ifnγ* (6 hpi)			
		*ifnγ* (12 hpi)			
		*ifnγ* (24 hpi)			

Level of expression is relative to time zero samples.

## Discussion

Cytology demonstrated the presence of LLCs with NCC and NK cell morphology. The size of zebrafish LLCs varied in size depending on their tissue locations, and similar variations in NK cell populations have also been observed in mammals [50]. In this study, we identified small agranular and large agranular LLCs in the livers, small agranular and granular and large agranular and granular LLCs in the kidneys and small agranular and granular and large granular LLCs in spleens of *rag1*^*-/-*^ mutant zebrafish. Two NK cell homologues have been described in teleosts: NCCs and NK-like cells (reviewed in [32]). Based on RT-PCR, lymphocyte-like cells from the *rag1*^*-/-*^ mutant zebrafish express NK cell lysin and NCCRP-1 [5]. NK lysin is expressed by NK cells [51], and NCCRP-1 is expressed by NCC cells [52], suggesting that NCCs and NK cells are included in the LCC populations we observed. The morphology of the larger zebrafish LLCs was similar to mammalian NK cells. The smaller LCCs we observed in our *rag1*^*-/-*^ mutant zebrafish were morphologically similar to zebrafish NCCs described by Moss *et al*. [53].

The expression of immune response genes *mx*, *tnfα*, *ifnγ*, *t-bet* (an NK cell transcription factor) and *nitr9* (a putative zebrafish NK cell receptor) in liver, kidney and spleen tissues support the presence of LLCs and demonstrate tissue specific differential responses of leukocytes following immune stimulation with TLR ligands. Fold changes are shown in Table 3.

β glucan did not affect *mx* expression in any *rag1*^*-/-*^ mutant zebrafish tissues, suggesting this ligand did not substantially induce type I interferons. Orally administered β glucan up-regulated *mx* expression 4 fold in liver tissue of common carp, but not in the head kidney or spleen [20]. Similar to studies in common carp [20], β glucan induced less than 10 fold increases (4 and 8 fold change) in kidney *tnfα* expression at 1 and 6 hpi in our *rag1*^*-/-*^ mutant zebrafish.

The overall greatest change following β glucan exposure was in *ifnγ* expression. β glucan stimulates a broad range of immune cell types, and this resulted in increased production of cytokines that further stimulated immune cells, resulting in an expanding immune response and additional accumulation of cytokines. Stimulated macrophages and dendritic cells further stimulate NK cells [54]. NK cells are the main producers of *ifnγ*, and we saw a 1500 fold increase of liver *ifnγ* expression at all time points in our mutant zebrafish (Table 3). Hepatic agranular small and large LLCs are associated with this dramatic expression. In mice, β glucan induced changes in cytokine expression correlated with changes in cell populations [55].

We found kidney *ifnγ* expression also increased (about 200 fold) following β glucan treatment, and large agranular and granular LLCs were associated with this expression. Similar increased *ifnγ* expression occurred in WT zebrafish kidney [56].

β glucan induced greater than 100 fold increases in splenic *ifnγ* expression at 6 and 24 hpi and between 10 and 100 fold increases at 1 and 12 hpi. Splenic leukocytes were 50% large granular LLCs and 27% small agranular LLCs. β glucan stimulates dendritic cells [57]. Dendritic cells are also present in zebrafish [58], and stimulated dendritic cells could be contributing to the changes in gene expression we observed.

β glucan induced a small increase in kidney *t-bet* expression, but did not induce changes in *t-bet* expression in any other tissues. Others found similar results in grass carp [59] and Atlantic salmon [60]. *T-bet* is a transcription factor for T cells and NK cells, and these findings suggest that in *rag1*^*-/-*^ mutant zebrafish, β glucan stimulated NK cell development in the kidney hematopoietic tissue, resulting in *t-bet* up-regulation that later decreased.

β glucan did not up-regulate *nitr9* expressions in any tissues in our mutant zebrafish. We saw decreased expression of *nitr9* at 12 and 24 hpi in the kidney tissue after β glucan treatment, relative to controls. It is interesting to note that *ifnγ* expression also decreased in the kidney relative to the peak level at these time points.

Poly I:C has been shown to be a strong inducer of interferon-inducible genes [32]. As an interferon inducible gene, *mx* expression is an indicator of increased type I interferon in fish [61] and mice [62]. In the liver, Poly I:C induced a <10 fold up-regulation of *mx* in our mutant zebrafish. Similar results were found in Atlantic salmon [63, 64].

We saw the greatest Poly I:C induction of *mx* expression increases (10 to 100 fold, Table 3) in kidney tissue. This was also seen by others in the kidney tissue of Poly I:C injected *rag1*^*-/-*^ zebrafish in microarray analysis [9]. Similar increases occurred in rainbow trout [65], Atlantic salmon and WT zebrafish kidney tissues [63, 66].

Poly I:C induced a <10 fold increase in splenic *mx* at 6 hpi and a 10 to 100 fold increase at 12 hpi. Similar increases were found in WT zebrafish spleen cells [66], and in rainbow trout [33], Atlantic salmon [67], and rock bream spleen tissues [68] and Poly I:C did not increase *mx* expression in carp spleen tissues [20].

In our study, Poly I:C did not affect liver *tnfα* and caused down regulation of *tnfα* in spleen and kidney tissues. Poly I:C had no effect on *tnfα* expression in common carp [20] and rainbow trout [33]. However, in gilthead seabream, Poly I:C significantly up-regulated *tnfa* in acidophilic granulocytes and macrophages at 0.5 and 1.5 hpi [69], and in the head kidney [70, 71] and rainbow trout anterior kidney leukocytes [72].

Poly I:C treatment had no effect on *ifnγ* expression in the liver and caused small inductions in the kidney (*ifnγ* up-regulated less than 10 fold (Table 3). In another study microarray analysis gave similar equivocal *ifnγ* change in kidney tissue of *Rag1*^*-/-*^ zebrafish [9]. Others documented induction of kidney *ifnγ* expression following Poly I:C treatment in Atlantic salmon [67], rainbow trout [42], grass carp [59], *Labeo rohita* [73], and WT zebrafish [66]. Poly I:C treatment had no effect on *ifnγ* expression in mutant zebrafish spleens. Similar results were found in Atlantic salmon [67]. In contrast, *ifnγ* was up-regulated in Poly I:C treated spleens in rainbow trout [42].

Poly I:C treatment had no effect on *t-bet* expression in the liver. However, it induced 1.2 fold up regulation of kidney *t-bet* expression. It also up-regulated kidney *t-bet* in WT zebrafish [66] and grass carp [59].

Following Poly I:C treatment, mutant zebrafish kidney tissue demonstrated a 19 fold increase in *nitr9* expression, suggesting stimulation of NK cells. This was the only Poly I:C induced up-regulation of *nitr9* in our study. Since *nitr9* is a putative receptor of zebrafish NK cells [44, 48], our findings suggest that Poly I:C induced kidney NK cell stimulation, and expansion of NK cells (demonstrated by kidney *t-bet* up-regulation).

R848 is immune modulating in mammals [74–76], Japanese flounder [77], Atlantic salmon [64], and rainbow trout [33, 72]. The NF-κB–My-d88 signaling pathway is conserved in fish, and was induced in Japanese flounder [77] after TLR 7/8 stimulation following R848 exposure. We found that R848 significantly up-regulated hepatic *mx* expression (50 fold increase), suggesting a steady rise in type I interferon expression in our mutant zebrafish. We also saw a 35 fold up-regulation of *mx* in kidney tissue. Increases in type 1 interferon were observed in rainbow trout [78] and Atlantic salmon kidney [64], and fluorescent *in* situ hybridization further demonstrated that a small population of head kidney cells produced type 1 interferon [64]. We also saw a 6 fold increase in splenic *mx* gene expression at 6 hpi and 8 fold increase at 12 hpi. In Atlantic salmon, R848 exposure caused type 1 interferon to be significantly up-regulated by a small population of splenic cells [64].

In our study, R848 did not induce differential *tnfα* expression in any tissues at any time. However, it induced up-regulation of *tnfα* in rainbow trout kidney [33], when used at levels higher than in our study.

The effects of R848 on *ifnγ*, *t-bet* or *nitr9* expressions are not documented in other fish. Gene expression does not necessarily correlate to protein expression, but western blots with Anti-NITR9^90.10.5^ antibody demonstrated increased protein expression after R848 treatment. We feel the significant increases seen in *t-bet* following TLR ligand treatments suggests that R848 likely resulted in an increased numbers of non-specific cytotoxic cells (NCCs) and NK cells, and changes in *nitr9* expression may reflect changes in the NK cell population.

## Conclusion

We identified LCCs that have characteristic NK cell morphology. Based on size, two LLC populations were found in the liver, kidney and spleen. Gene expression data supported the observations of resident LCC populations in the liver, spleen and kidney.

β glucan treatment caused broad changes in gene expression, predominately *ifnγ*, and *tnfα*. Tnfα is a strong pro-inflammatory cytokine produced predominately from stimulated macrophages and *ifnγ* is produced by stimulated NK cells and macrophages. Because of this strong proinflammatory environment, the direct effect of β glucan on gene expression is difficult to interpret. β glucan did not induce changes in the NK cell specific genes t*-bet* or *nitr9* in the liver or spleen.

Poly I:C induced significant *mx* up-regulation, which is indicative of type 1 interferons. Type 1 interferons induce multiple immune changes, and probably induced the *tnfα* down regulation observed in our zebrafish kidney tissue. Significant increases in *t-bet* and *nitr9* suggest NK cell expansion or differentiation and stimulation. In lymphocyte deficient animals, *ifnγ* is primarily produced by stimulated NK cells and by macrophages to a lesser extent. This study demonstrates that Poly I:C produces a more limited and possibly more focused response with type I interferon and NK cell stimulation but limited macrophage stimulation.

Following R848 treatment, the greatest up-regulation of *nitr9* and *t-*bet occurred in the liver. R848 also induced NK cell stimulation and possible NK cell expansion in the kidney. Our studies suggest R848 stimulates NK cells better than Poly I:C, resulting in increased *t-bet*, *nitr9* and *ifnγ* expression. R848 is a better NK cell inducer than Poly I:C in humans as well [31].

Overall, the most substantial and rapid up regulation of a gene expression was hepatic *ifnγ*. In comparison, changes in splenic immune gene expressions were much lower than in the liver and kidney. None of the ligands induced *t-bet* and *nitr9* expression at any time point in this tissue, suggesting that under the conditions of this study, splenic LLCs were not stimulated to differentiate, respond immunologically, or proliferate.

*T-bet* up-regulation either preceded or occurred simultaneously with *nitr9*, and changes in *nitr9* expression never preceded those of *t-bet*. This suggests that NK cell expansion and differentiation may occur before increased *nitr9* expression. NK cell stimulation and expansion was documented in T and B cell deficient rag2^*-/-*^ mutant mice upon exposure and re-exposure to murine cytomegalovirus [79]. Zebrafish LLCs may be able to respond similarly.

Our finding of β glucan induction of macrophages mirrors findings in mice resulting from macrophage training [80, 81]. In summary, these findings suggest that both NK cell based immunity and macrophage training may occur in T and B cell deficient *rag1*^*-/-*^ mutant zebrafish following TLR ligand exposure and this may explain protection seen in pathogen challenges [6, 9]. Dendritic cells may also be involved in a manner not yet discovered. In future studies, we plan to evaluate the influence of these ligands on protection using the ESC/ *rag1*^*-/-*^ mutant zebrafish model.

## Supporting information

S1 Fig Expression analyses of *mx*, *tnfα*, *ifnγ*, *t-bet* and *nitr9* in liver, kidney and spleen of non-injected (control) adult *rag1*^*-/-*^ mutant zebrafish (n = 6) were analyzed by RT-qPCR. Gene expression levels of *mx*, *tnfα*, *ifnγ*, *t-bet* and *nitr9* were normalized with housekeeping gene, *arp*, expression levels. No significant differences were observed in the gene expressions between tissues prior to injecting *rag1*^*-/-*^ mutant zebrafish with TLR ligands. Data are presented as mean fold change ± standard deviation.
(PDF)

S1 Table Differential immune gene expression following stimulation of toll like receptor (TLR) ligands in *rag1*^*-/-*^ mutant zebrafish. The numbers highlighted in grey denote statistical significance.
(PDF)
