# Supplementary figures and images for: Differential gene expression following TLR stimulation in rag1-/- mutant zebrafish tissues and morphological descriptions of lymphocyte-like cell populations
Source: PLoS One. 2017 Sep 14;12(9):e0184077. doi: 10.1371/journal.pone.0184077 (PMC5598945; doi:10.1371/journal.pone.0184077)

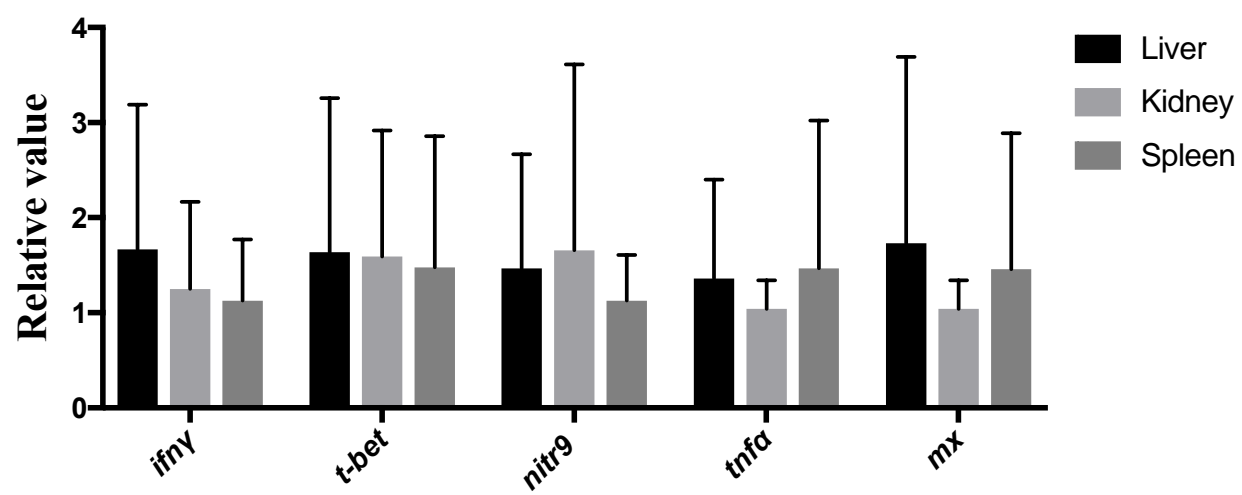

Supplement: S1 Fig — Gene expression levels of mx, tnfα, ifnγ, t-bet and nitr9 were normalized with housekeeping gene, arp, expression levels. No significant differences were observed in the gene expressions between tissues prior to injecting rag1-/- mutant zebrafish with TLR ligands. Data are presented as mean fold change ± standard deviation. (PDF) [file pone.0184077.s001.pdf]
