# Supplementary material for: Differential gene expression following TLR stimulation in rag1-/- mutant zebrafish tissues and morphological descriptions of lymphocyte-like cell populations
Source: PLoS One. 2017 Sep 14;12(9):e0184077. doi: 10.1371/journal.pone.0184077 (PMC5598945; doi:10.1371/journal.pone.0184077)
Supplement: S1 Table — The numbers highlighted in grey denote statistical significance. (PDF) [file pone.0184077.s002.pdf]

Treatment	Gene	Tissue	Time	p value	Mean ± SD (Treatment)	Mean ± SD (PBS)
**β glucan** (Fig 5)	***mx***	Liver	All time points	0.7871		
		Kidney	All time points	0.1386		
		Spleen	All time points	0.2652		
	tnfα	Liver (Fig 5A)	1 hpi	0.0122	9.97±3.09	1.14±0.78
			6 hpi	0.0802	4.77±1.33	0.91±0.08
			12 hpi	0.0415	13.55±4.41	3.55±1.03
			24 hpi	0.9427	4.71±3.03	6.21±1.77
		Kidney (Fig 5B)	1 hpi	0.0006	4.35±1.15	0.25±0.14
			6 hpi	0.0002	8.34±0.53	0.21±0.04
			12 hpi	0.0463	1.99±1.38	0.29±0.07
			24 hpi	0.7631	3.74±1.36	0.61±0.92
		Spleen	All time points	0.2386		
	ifnγ	Liver (Fig 5C)	1 hpi	<0.0001	1502.27±300.76	167.53±122.24
			6 hpi	<0.0001	1226.20±518.96	115.95±11.93
			12 hpi	<0.0001	1741.50±904.84	207.35±103.17
			24 hpi	<0.0001	2232.38±966.22	578.55±225.71
		Kidney (Fig 5D)	1 hpi	0.0004	68.61±27.67	7.05±3.89
			6 hpi	<0.0001	193.93±81.42	9.63 ±4.98
			12 hpi	0.0072	62.57±20.94	12.63±4.59
			24 hpi	0.4280	96.18±28.98	59.48±49.36
		Spleen (Fig 5E)	1 hpi	<0.0001	70.24±30.89	13.40±8.90
			6 hpi	<0.0001	110.87±18.95	8.38±3.71
			12 hpi	<0.0001	94.85±30.77	31.10±26.71
			24 hpi	<0.0001	116.27±36.22	44.98±12.16
	t-bet	Liver	All time points	0.4319		
		Kidney (Fig 5F)	1 hpi	0.5820	0.24±0.07	0.35±0.17
			6 hpi	0.0332	0.59±0.19	0.28±0.09
			12 hpi	0.9899	0.28±0.07	0.31±0.05
			24 hpi	0.7258	0.35±0.07	0.47±0.16

		Spleen	All time points	0.1656		
	nitr9	Liver	All time points	0.3400		
		Kidney (Fig 5G)	1 hpi	0.9361	1.31±1.24	1.48±0.40
			6 hpi	0.8726	1.30±0.05	1.10±0.36
			12 hpi	0.0062	4.17±0.86	5.12±0.84
			24 hpi	<0.0001	1.89±1.46	7.1±1.03
		Spleen	All time points	0.1060		
**Poly I:C** (Fig 6)	***mx***	Liver (Fig 6A)	1 hpi	0.0145	3.80±0.98	2.12±1.06
			6 hpi	0.0014	17.01±7.64	2.33±1.26
			12 hp	<0.0001	23.27±9.71	2.78±1.48
			24 hpi	0.0002	14.50±7.93	3.11±1.50
		Kidney (Fig 6B)	1 hpi	0.9973	3.19±1.52	3.07±2.35
			6 hpi	0.0182	19.59±15.54	1.86±1.53
			12 hpi	0.0003	40.86±24.64	1.09±0.44
			24 hpi	0.0052	13.04±5.56	1.08±0.48
		Spleen (Fig 6C)	1 hpi	0.9999	3.15±1.22	2.60±0.36
			6 hpi	0.0028	8.03±5.67	0.13±0.18
			12 hpi	0.0107	19.76±9.02	0.82±0.44
			24 hpi	0.3500	5.95±3.46	1.42±1.07
	tnfα	Liver	All time points	0.1843		
		Kidney (Fig 6D)	1 hpi	0.9997	0.97±0.26	1.12±0.36
			6 hpi	0.9783	3.51±2.87	1.00±0.75
			12 hpi	0.4310	1.77±0.42	0.61±0.37
			24 hpi	0.0194	0.17±0.02	0.67±0.24
		Spleen	1 hpi	0.9829	4.01±0.69	3.19±0.19
			6 hpi	0.9894	1.59±1.00	1.09±0.48
			12 hpi	0.4594	0.59±0.18	2.38±0.98
			24 hpi	0.0041	0.17±0.03	2.13±0.55
	ifnγ	Liver	All time points	0.3992		
		Kidney (Fig 6F)	1 hpi	0.9090	4.22±1.20	2.81±0.13
			6 hpi	0.6046	8.38±4.31	4.59±2.68

			12 hpi	0.0025	6.31±2.14	0.99±0.95
			24 hpi	0.9973	2.76±2.38	2.57±0.64
		Spleen	All time points	0.0521		
	t-bet	Liver	All time points	0.1279		
		Kidney (Fig 6G)	1 hpi	0.2044	0.58±0.20	0.35±0.17
			6 hpi	0.0004	1.21±0.30	0.28±0.09
			12 hpi	0.0913	0.64±0.26	0.31±0.05
			24 hpi	0.9978	0.44±0.13	0.47±0.16
		Spleen	All time points	0.1470		
	nitr9	Liver	All time points	0.5372		
		Kidney (Fig 6H)	1 hpi	0.0274	18.77±7.86	1.13±0.52
			6 hpi	0.7303	2.09±1.62	0.58±0.19
			12 hpi	0.7253	1.23±1.13	2.69±1.49
			24 hpi	0.2842	1.09±1.00	3.77±0.54
		Spleen	All time points	0.1954		
**R848** (Fig 7)	***mx***	Liver (Fig 7A)	1 hpi	0.0003	11.79±3.23	2.12±1.06
			6 hpi	0.0002	30.46±12.59	2.33±1.26
			12 hpi	<0.0001	26.04±10.29	2.78±1.48
			24 hpi	<0.0001	58.25±20.03	3.11±1.50
		Kidney (Fig 7B)	1 hpi	0.0009	11.41±9.57	3.07±2.35
			6 hpi	0.0008	32.60±10.69	1.86±1.53
			12 hpi	0.0052	13.74±9.12	1.09±0.44
			24 hpi	0.0014	19.03±9.75	1.08±0.48
		Spleen (Fig 7C)	1 hpi	0.6934	5.67±1.05	2.60±0.36
			6 hpi	<0.0001	4.37±1.20	0.06±0.03
			12 hpi	0.0137	7.73±2.56	0.82±0.44
			24 hpi	0.2371	4.70± 2.43	1.42 ±1.07
	tnfα	Liver	All time points	0.1815		
		Kidney	All time points	0.4432		
		Spleen	All time points	0.1167		
	ifnγ	Liver (Fig 7D)	1 hpi	0.9897	16.27±5.22	29.08±24.58

			6 hpi	0.0310	100.37±40.04	8.85±2.37
			12 hpi	0.0040	65.41±32.45	2.49±2.24
			24 hpi	0.0007	41.83±21.16	1.10±0.90
		Kidney (Fig 7E)	1 hpi	<0.0001	12.61±4.14	2.81±0.13
			6 hpi	<0.0001	153.23±90.34	4.59±2.68
			12 hpi	0.0030	31.45±25.55	0.99±0.95
			24 hpi	<0.0001	24.34±15.29	2.57±0.64
		Spleen (Fig 7F)	1 hpi	0.7600	2.13±1.24	3.46±0.47
			6 hpi	0.0229	6.37±2.12	0.90±0.19
			12 hpi	0.0179	8.35±3.22	0.70±0.30
			24 hpi	0.0055	5.54±1.78	0.87±0.66
	t-bet	Liver (Fig 7G)	1 hpi	<0.0001	106.64±77.90	1.29±0.68
			6 hpi	0.0006	11.69±5.48	1.41±0.28
			12 hpi	0.9699	2.16±0.88	2.71±1.07
			24 hpi	<0.0001	179.29±84.28	3.85±1.56
		Kidney (Fig 7H)	1 hpi	0.0006	3.80±2.80	0.35±0.17
			6 hpi	0.0006	7.15±1.99	0.28±0.09
			12 hpi	0.9913	0.68±0.80	0.31±0.05
			24 hpi	0.0789	2.47±1.17	0.47±0.16
		Spleen	All time points	0.0950		
	nitr9	Liver (Fig 7I)	1 hpi	<0.0001	49.13±27.15	0.51±0.31
			6 hpi	0.0002	6.22±2.76	0.42±0.35
			12 hpi	0.6616	0.97±0.12	0.56±0.07
			24 hpi	0.9573	3.11±1.64	2.53±1.74
		Kidney (Fig 7J)	1 hpi	0.2292	0.90±0.56	0.77±0.73
			6 hpi	0.0010	10.18±2.59	0.58±0.19
			12 hpi	0.0811	0.51±0.15	2.69±1.49
			24 hpi	0.1154	0.91±0.30	3.77±0.54
		Spleen	All time points	0.4584		
